# The effect of antiretroviral therapy initiation on vitamin D levels and four oral diseases among Kenyan children and adolescents living with HIV

**DOI:** 10.1371/journal.pone.0275663

**Published:** 2022-10-13

**Authors:** Ana Lucia Seminario, Arthur Kemoli, Walter Fuentes, Yan Wang, Poojashree Rajanbabu, Dalton Wamalwa, Sarah Benki-Nugent, Grace John-Stewart, Jennifer A. Slyker

**Affiliations:** 1 Department of Pediatric Dentistry, University of Washington, Seattle, Washington, United States of America; 2 Department of Global Health, University of Washington, Seattle, Washington, United States of America; 3 Department of Pediatric Dentistry & Orthodontics, University of Nairobi, Nairobi, Kenya; 4 Petaluma Health Center, University of California San Francisco, Petaluma, California, United States of America; 5 Division of Infectious Diseases, Department of Medicine, University of California Los Angeles, Los Angeles, California, United States of America; 6 University of Washington Timothy A. DeRouen Center for Global Oral Health, Seattle, Washington, United States of America; 7 Department of Paediatrics and Child Health, University of Nairobi, Nairobi, Kenya; 8 Department of Pediatrics and Department of Medicine, University of Washington, Seattle, Washington, United States of America; 9 Department of Epidemiology, University of Washington, Seattle, Washington, United States of America; Indiana University School of Medicine, UNITED STATES

## Abstract

**Objectives:**

The impact of antiretroviral treatment (ART) on the occurrence of oral diseases among children and adolescents living with HIV (CALHIV) is poorly understood. The aim of this study was to determine the effect of ART timing on vitamin D levels and the prevalence of four oral diseases (dry mouth, dental caries, enamel hypoplasia, and non-herpes oral ulcer) among Kenyan CALHIV from two pediatric HIV cohorts.

**Methods:**

This nested cross-sectional study was conducted at the Kenyatta National Hospital, Nairobi, Kenya. CALHIV, 51 with early-ART initiated at <12 months of age and 27 with late-ART initiated between 18 months-12 years of age, were included. Demographics, HIV diagnosis, baseline CD4 and HIV RNA viral load data were extracted from the primary study databases. Community Oral Health Officers performed oral health examinations following standardized training.

**Results:**

Among 78 CALHIV in the study, median age at the time of the oral examination was 11.4 years old and median ART duration at the time of oral examination was 11 years (IQR: 10.1, 13.4). Mean serum vitamin D level was significantly higher among the early-ART group than the late-ART group (29.5 versus 22.4 ng/mL, p = 0.0002). Children who received early-ART had a 70% reduction in risk of inadequate vitamin D level (<20 ng/mL), compared to those who received late-ART (p = 0.02). Although both groups had similar prevalence of oral diseases overall (early-ART 82.4%; late-ART 85.2%; p = 0.2), there was a trend for higher prevalence of dry mouth (p = 0.1) and dental caries (p = 0.1) in the early versus late ART groups. The prevalence of the four oral diseases was not associated with vitamin D levels (p = 0.583).

**Conclusions:**

After >10 years of ART, CALHIV with early-ART initiation had higher serum vitamin D levels compared to the late-ART group. The four oral diseases were not significantly associated with timing of ART initiation or serum vitamin D concentrations in this cohort. There was a trend for higher prevalence of dry mouth and dental caries in the early-ART group, probably as side-effects of ART.

## Introduction

Oral diseases constitute a significant public health burden that disproportionally affects the quality of life for vulnerable populations, including children and adolescents living with HIV (CALHIV) [1–3]. Studies analyzing how antiretroviral treatment (ART) exposure influences the occurrence of oral diseases among CALHIV are limited [4]. Perinatally HIV-infected youth participating in the Pediatric HIV/AIDS Cohort Study had a high prevalence of dental caries and periodontal disease despite ART [5]. A follow-up study in 2018 showed that 61% of perinatally HIV-infected youth had untreated dental caries, and those with later initiation of combination ART had a significantly higher number of teeth affected (decay, missing, filled teeth—DMFT) and of surfaces within each tooth (decay, missing, filled surfaces -DMFS) than those whose initiation to ART was at a younger age [6].

In countries with high HIV prevalence, there is a need to investigate preventive approaches to decrease the high occurrence of dental co-morbidities among PWH. One potential intervention is vitamin D supplementation. Recent findings suggest that this pro-hormone might play a role in deaccelerating the progression of oral diseases. Possible mechanisms of vitamin D deficiency include decreasing the saliva flow and altering saliva composition by reducing the amount of calcium ions and pH; altering normal tooth development and mineralization process in utero, specifically during the second and third trimester, generating dental hypoplasias and consequently increasing the risk for dental caries; decreasing the activation of cathelicidins and defensins–antimicrobial peptides (AMPs) present in lysosomes of macrophages and leukocytes and increased dentinal remineralization and dentin formation in response to a dental caries attack [7–11]. Vitamin D supplementation increases salivary AMPs production in adults [12–14], and could be useful in preventing oral disease.

Data on vitamin D and oral health in children is scarce. In Kenya, serum vitamin D deficiency is highly prevalent among urban children due to malnourishment and limited sun exposure [15]. Additionally, people with darker skin do not achieve optimal serum vitamin D concentrations because pigmentation reduces vitamin D production in the skin [16]. There are high levels of oral diseases in children living with HIV in Africa. Among 307 (12 year old children living with HIV from West Africa), 84.6% experienced caries, with a mean DMFS of 11.6 and a mean DMFT of 5.0 [17]. Describing levels of vitamin D and occurrence of oral diseases in CALHIV in the context of ART will inform the development of potential interventions to reduce the burden of oral diseases in CALHIV. In a cohort of CALHIV in Nairobi, Kenya we evaluated the influence of ART timing on vitamin D levels and four oral diseases.

## Methods

This cross-sectional study was approved by Institutional Review Board at the University of Washington (STUDY00003298) and by the Kenyatta National Hospital/University of Nairobi Ethics Research Committee (KNH/ERC/R/133). Participants were enrolled and underwent all study procedures at the Kenyatta National Hospital in Nairobi, Kenya.

### Study population: The Kenyan pediatric studies

The study was nested into longitudinal cohorts of Kenyan CALHIV followed since initiation of ART (Kenya Pediatric Studies (KPS)). Children were initially enrolled into two clinical trials focused on optimizing care and treatment for CALHIV [18, 19]. The first one was the Optimizing Pediatric HIV-1 Treatment study (OPH). This randomized clinical trial (2007–2010) enrolled children <12 months old with immediate ART initiation; a planned treatment interruption was conducted at 24 months post-ART to test for spontaneous viral control (NCT00428116) [18]. The second cfohort was the Pediatric Adherence Study (PAD) that enrolled children >18 months to 12 years old from 2004–2005 who met contemporaneous ART criteria (*moderate (World Health Organization (WHO) clinical stage 2 with CD4 <15%) to severe (WHO clinical stage 3 or 4) HIV-1 disease)* [20, 21]. All ART regimens were selected in accordance with Kenyan national guidelines. Study visits were conducted at 3 to 6 monthly intervals, with administration of a standardized questionnaire and blood collection. Clinical data, adherence and ART information, HIV RNA level, and CD4 counts were collected at each visit.

### Recruitment

Study Clinical Officers recruited KPS families to participate in this study of oral diseases. Caregivers provided written informed consent for participation, and children over 8 years old were additionally asked to assent to study procedures. Study information and written consents were available in both English and Kiswahili, allowing families to select their preferred language. All KPS staff were fluent in both languages and have an established trust with the KPS families.

### Data collection

Demographics, HIV diagnosis, baseline CD4 and HIV RNA viral load data were extracted from the parent study databases. Community Oral Health Officers (COHOs) performed oral health examinations with dental mirrors, using natural light augmented with a head lamp and the patient lying on a medical bed. The COHOs were trained and calibrated in the diagnosis of dental defects and oral manifestations of HIV, such as oral candidiasis, angular cheilitis, HSV ulcers, aphthous ulcers, parotid enlargement, and ulcerative gingivitis. Calibration for diagnosing oral manifestations of HIV and dental defects was done following validated training modules provided by the University of Washington and University of California-San Francisco [22]. The calibration was tested for accuracy showed an inter-rater reliability score of 0.78 and an intra-rater reliability of 0.85. Calibrated Kenyan COHOs conducted oral examinations during routine KPS study visits. All examinations occurred onsite in a room adjacent to the HIV clinic. Due to the lack of infrastructure, we will not take dental radiographs.

### Oral health endpoints

Oral examinations were conducted between February to August of 2019. The evaluation of HIV-associated oral mucosal lesions was performed in accordance with the WHO Oral Health Surveys and Record Form for Oral Manifestations of HIV/AIDS [23]. For dental caries, teeth were dried with gauze, visual assessment was conducted, and lesions (cavitated and white spots) [24] were recorded. Dental caries was assessed by presence (Y/N) and calculated in terms of the number of decayed teeth, missing teeth due to caries, and filled teeth (DMFT for permanent dentition and dmft for primary dentition). The DMFT index depicts previous and current dental disease and also provides details on the severity of the caries in a child, as it reflects the number of teeth involved. For this study, we analyzed four most prevalent oral diseases in the cohort, to determine a possible association to vitamin D deficiency: Ulcer (not HSV/aphthous), dry mouth (xerostomia) due to decreased salivary flow, enamel hypoplasia and dental caries [25–27].

### Vitamin D assessment

As part of the parent study procedures, CALHIV had blood collected every 6 months. The oral health assessments were scheduled to occur on the same day as the children’s next venipuncture appointment. Blood specimens were collected from all participants for serum 25-hydroxyvitamin D (vitamin D) analysis. Briefly, 5 ml of venous blood was collected and transported on dry ice to the study laboratory. Serum was removed and stored at -80°C until analysis using chemiluminescent immunoassay (CLIA) technology. Samples were analyzed with appropriate standards, deuterated internal standards, and quality control using the LIAISON platform (DiaSorin S.p.A, Sallugia, VC, Italy). Plasma 25(OH)D concentration was categorized as adequate (≥20ng/mL) and inadequate (<20ng/mL) [28], based on the Endocrine Society Clinical Practice Guidelines and the US Institute of Medicine [29].

### Clinical data

Clinical data and HIV labs were extracted from the KPS database. HIV RNA viremia was defined as >400 copies/mL. CD4 cell count data at the time of the dental exam was abstracted.

### Data management and statistical analysis

Data from the oral health examination was recorded on paper forms and entered into a Research Electronic Data Capture (REDCap) database (Institute of Translational Health Sciences). Mean/standard deviation and frequency/percentage were used to describe the characteristics of the overall study population and stratified by early-ART (OPH Cohort) or late-ART (PAD Cohort) timing of ART initiation. The two-sample t-test was used to compare mean vitamin D levels and dmft/DMFT by early/late-ART initiation, and Fisher’s exact test was used to compare the prevalence of the four oral diseases by vitamin D levels with by timing of ART initiation. We applied the Poisson regression model with cohort (early/late-ART) as clustered effect to estimate the risk ratio with and without adjust for age at the time of oral exam [30, 31]. The *proc genmod* procedure in SAS 9.4 with a robust error variance estimation was utilized for all analyses, and all analyses used 2-tailed tests with alpha = 0.05.

## Results

### Cohort characteristics

A total of 78 children participated in the study (none of the caregivers declined to participate), 51 (65.4%) received early-ART in the first year of life and 27 (34.6%) were enrolled at an older age and started ART per clinical criteria. Approximately half of the children were male (52.6%), with mixed dentition (65.4%) and lived in families where the average number of children were 2.0 (IQR: 1.0, 2.5). The median age of the children overall was 11.4 years (IQR: 10.7, 16.2) at the oral exam; 10.8 years (IQR: 10.4, 11.3) in the early-ART group and 17.1 years (IQR: 16.0, 19.2) in the late-ART group, respectively.

In the early-ART group, 56.9% received non-nucleoside reverse transcriptase inhibitor-(NNRTI)-based and 43.1% received protease-inhibitor (PI)-based first-line ART regimens. In the late-ART group, all received NNRTI-based regimens [18, 32]. Median ART duration at the time of oral examination overall was 11 years (IQR: 10.1, 13.4); 10.4 years (IQR: 9.9, 11.0) in the early-ART group and 13.9 years (IQR: 12.9, 14.3) in the late-ART group. A few (31.4%) early-ART children had participated in the randomized trial of treatment interruption (TI), with median duration of TI in these participants of 4.5 months (IQR 3.5–11.6). Median enrollment CD4 count at the oral exam was 954 cells/mm^3^ (IQR: 681, 1,286). For caregivers, the majority were female (87.2%) with a median of 10 years of education (IQR: 8, 12) (Table 1).

**Table 1:** Characteristics of children included in the oral diseases study.

Variables	Combined cohort	Early (OPH cohort)	Late (PAD cohort)
	(n = 78)	(n = 51)	(n = 27)
	**Median (IQR) or N (%)**		
**Age (years)**			
** At enrollment**	1 (0.4,2.9)	0.4 (0.3,1)	3.5 (2.6,5.1)
** At oral exam**	11.4 (10.7,16.2)	10.8 (10.4,11.3)	17.1 (16,19.2)
**Sex**			
** Female**	37 (47.4)	22 (43.1)	15 (55.6)
**Dentition Status**			
** Mixed Dentition**	51 (65.4)	51 (100)	0 (0)
** Permanent Dentition**	27 (34.6)	(0)	27 (100)
**Years on ART**	11 (10.1,13.4)	10.4 (9.9,11)	13.9 (12.9,14.3)
**First-line ART regimen**			
NNRTI-based regimen*	56(71.8)	29(56.9)	27(100)
PI-based regimen**	22(28.2)	22(43.1)	0 (0)
**Randomized to TI** ***	4.5(3.5,11.6)	4.5(3.5,11.6)	NA
**CD4 Count (cells/mm^3^)**			
** At ART initiation**	1028 (516,1572)	1326 (735,1757)	522 (102,982)
** At oral exam**	954 (681,1286)	1059 (749,1370)	764 (533,1269)
**Caregiver age (years)**	39.5 (34,44)	37 (33,43)	41.5 (37.5,44)
**Caregiver sex**			
** Female**	68 (87.2)	47 (92.2)	21 (77.8)
** Male**	6 (7.7)	3 (5.9)	3 (11.1)
** Not reported**	4 (5.1)	1 (2)	3 (11.1)
**Caregiver education (years)**	10 (8,12)	10 (8,12)	10 (8,12)
**Number of children per family**	2 (1,2.5)	2 (1,3)	1 (1,2)

*Nevirapine or efavirenz-based regimens,

**Lopinavir/ritonavir,

***TI = structured treatment interruption in the OPH cohort

### Vitamin D levels and oral diseases in children with early/late-ART initiation

**Fig 1:**
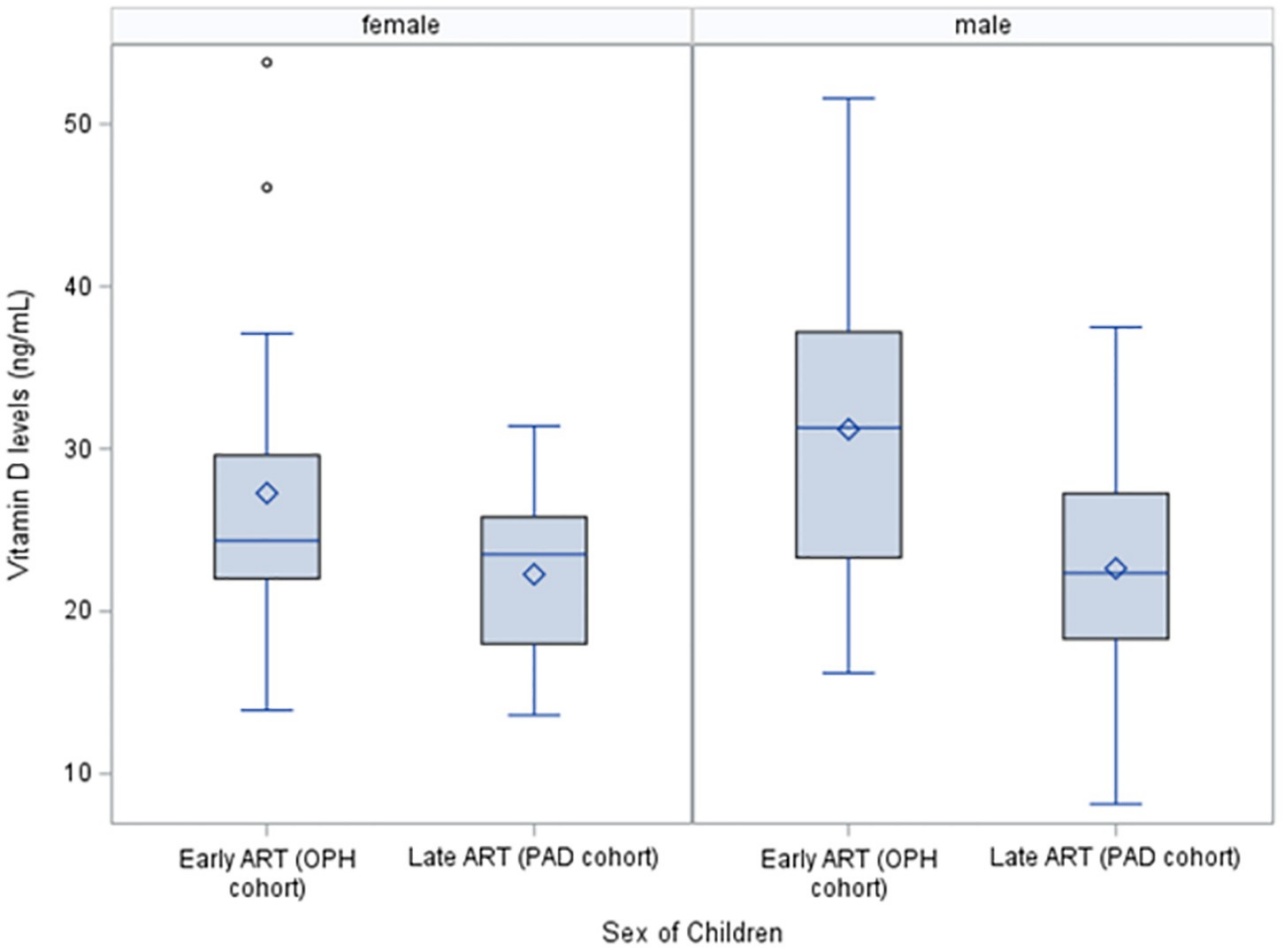
Box plot of vitamin D level (ng/ml) by early- and late-ART initiation and by sex of children. The middle line inside of the box is the median. The diamond inside of the box is the mean. The box represents the interquartile range (IQR, Q1 to Q3, from 25^th^ percentile to 75^th^ percentile). The upper and lower bars represent the Q1-1.5*IQR and Q3+1.5*IQR. The circles outside the bar could be potential outliers.

About 18% of the children had inadequate serum vitamin D levels (<20ng/mL), and inadequate vitamin D levels were more prevalent among the late-ART group (33.3%) compared to the early-ART group (9.8%) (p = 0.01). Mean serum vitamin D level was 22.4 ng/mL for late-ART and 29.5 ng/mL for early-ART (p = 0.0002) (Table 2). Because sex hormones have been associated with serum vitamin D levels in previous studies [33, 34], we additionally compared vitamin D levels stratified by sex assigned at birth and found that among females, the early-ART group had a trend for a higher mean vitamin D level compared to the late-ART group (27 ng/mL vs. 24 ng/mL, p = 0.12), and among males, the early-ART group also had a higher mean vitamin D level compared to the late-ART group (31 ng/mL vs. 22 ng/mL, p = 0.01) (Fig 1). In a stratified analysis of the early-ART group, those who received PI-based first-line regimens had a higher vitamin D level (30.9 vs. 28.4, p = 0.35) as those who received NNRTI-based first-line regimens.

**Table 2:** Levels of vitamin D and oral diseases by ART initiation time.

	All children	Early-ART (OPH)	Late-ART (PAD)	P-value*
**Characteristics**	**Mean ± SD or N (%)**			
**Vitamin D level (ng/mL)**	27.1 ± 9.1	29.5 ± 9.3	22.4 ± 6.4	0.0002^b^
**Vitamin D categories**				
** Adequate (≥ 20 ng/mL)**	64 (82.1)	46 (90.2)	18 (66.7)	0.01^a^
** Inadequate (< 20 ng/mL)**	14 (17.9)	5 (9.8)	9 (33.3)	
**Oral Diseases**				
** Any Oral Disease**	65 (83.3)	42 (82.4)	23 (85.2)	0.2^a^
** Dental Caries**	53 (68)	37 (72.6)	16 (59.3)	0.1^a^
** Dry Mouth**	23 (29.5)	17 (33.3)	6 (22.2)	0.1^a^
** Enamel Hypoplasia**	7 (9)	6 (11.8)	1 (3.7)	0.2^a^
** Other Ulcer (not HSV/aphthous)**	5 (6.4)	4 (7.8)	1 (3.7)	0.3^a^
**Number of decayed, missing (due to caries), and filled teeth DMFT/dmft**	2.6 ± 2.6	2.5 ± 2.4	2.8 ± 3	0.7^b^

*Calculated using Fisher’s exact test^a^ and two-sample t-test^b^

The four oral diseases were observed in the majority of CALHIV (83.3%) (Table 2). Children with early- and late-ART initiation had a similar prevalence of oral diseases overall (early-ART 82.4%; late-ART 85.2%; p = 0.2), with dry mouth being the most common oral lesion observed (29.5% overall) with a trend for higher prevalence among early-ART group (early-ART 33.3%; late-ART 22.2%; p = 0.1). Dental caries affected 68.0% of the CALHIV overall. While trends for a higher prevalence of dental caries, enamel hypoplasia, and other ulcers (not HSV or Aphthous ulcers) were observed in the early-ART group, these values did not reach significance at p<0.05. Caries experiences measured by DMFT/dmft were also not significantly different between groups (Table 2).

### Correlates of inadequate vitamin D status

Children with early-ART initiation had a 70% reduction in risk of inadequate vitamin D level (<20ng/mL), compared to those with late-ART initiation (Table 3, p = 0.02). CALHIV who were 15 years or younger had a 70% reduction in risk of inadequate vitamin D, compared to those who were older than 15 years of age (p = 0.01), likely reflecting both age and early-ART given the significantly younger age of the early-ART group. Prevalence of inadequate vitamin D level did not significantly differ by sex of caregiver (p = 0.93), caregiver’s education level (higher than primary (8 years of education and above) or not) (p = 0.36), whether the number of children per family was greater than 2 (p = 0.79) or whether CD4 count was higher than 500 cells/ml (p = 0.42) (Table 3).

**Table 3:** Association between vitamin D status (inadequate) and characteristics of Kenyan children on long-term ART (Risk Ratio (RR) and 95% confidence intervals (CI), both unadjusted and adjusted by age and sex).

Characteristics	% Vitamin D Inadequate	Unadjusted RR	p-value	Adjusted RR^b^	p-value
**Cohort**					
** Early-ART**	9.8%	0.3 (0.1,0.8)	0.0153	0.2 (0,1.4)	0.0995
** Late-ART**	33.3%				
**Age** ^a^					
** < = 15**	9.6%	0.3 (0.1,0.7)	0.0109		
** >15**	34.6%				
**Child sex**					
** Male**	12.2%	0.5 (0.2,1.4)	0.1755		
** Female**	24.3%				
**Caregiver sex**					
** Male**	16.7%	0.9 (0.1,5.9)	0.9326	0.9 (0.2,3.6)	0.8814
** Female**	19.1%				
**Education**					
** 8+ years**	20.3%	1.9 (0.5,7.9)	0.3581	2 (0.5,8.1)	0.3074
** <8 year**	10.5%				
**Number of child**					
** < = 2**	18.5%	1.2 (0.3,4.7)	0.7947	1 (0.2,4.3)	0.9842
** >2**	15.4%				
**CD4 counts**					
** > = 500**	16.9%	0.6 (0.2,2.1)	0.4214	0.8 (0.2,2.7)	0.7062
** <500**	28.6%				
**Regimen**					
** NNRTI**	19.6%	0.7 (0.2,2.3)	0.5435	1.5 (0.4,6.4)	0.5684
** PI-based**	13.6%				

^a^Cutoff age for adulthood in Kenya [35, 36]

^b^Adjusted for age and sex at the time of oral exam

When adjusting for age and sex at assessment, the effect size of the protective association between early-ART and inadequate vitamin D became stronger; those receiving early-ART had an 80% reduction in risk of inadequate vitamin D level (CI = 0.0,1.4; p = 0.01). Point estimates for all other covariates were similar when controlling for age at assessment.

### Vitamin D and oral diseases

In a Poisson regression model with early- and late-ART cohorts as clustered effects, inadequate vitamin D levels were not associated with any oral diseases assessed: untreated dental caries, dry mouth, or enamel hypoplasia (Table 4). Associations did not change with or without controlling for age at the time of oral exam.

**Table 4:** Associations between serum vitamin D and oral diseases (Risk Ratio (RR^a^) and 95% confidence intervals (CI), both unadjusted and adjusted by age).

Oral disease	Vitamin D level		Unadjusted RR		Age adjusted RR	
Any oral disease	Mean ± Std	Inadequate %	Risk ratio (RR)	p-value	adjusted RR	p-value
** Yes**	27.3 ± 8.5	16.9%	0.9 (0.7,1.2)	0.6338	0.9 (0.7,1.2)	0.5834
** No**	26.1 ± 12	23.1%				
Dental caries						
** Yes**	28.1 ± 8.8	15.1%	0.8 (0.5,1.3)	0.3978	0.8 (0.5,1.4)	0.4743
** No**	24.8 ± 9.4	24%				
Dry mouth						
** Yes**	27 ± 6.6	17.4%	1 (0.4,2.4)	0.9342	1.1 (0.4,2.8)	0.8589
** No**	27.1 ± 10	18.2%				
Enamel hypoplasia						
** Yes**	29.7 ± 7	14.3%	0.8 (0.1,5.8)	0.7935	1 (0.1,8.9)	0.971
** No**	26.8 ± 9.2	18.3%				
Other ulcer (not HSV/aphthous)						
** Yes**	26.9 ± 3.3	0%				
** No**	27.1 ± 9.3	19.2%				

^a^RR was estimated by Poisson regression model, with early/late cohort as clustered effect. Both RR with and without age adjusted were reported in the table.

## Discussion

In this nested cross-sectional study, we assessed the influence of ART initiation timing on vitamin D serum levels and four oral diseases among Kenyan CALHIV. We found a high prevalence of inadequate serum vitamin D levels, and CALHIV with early-ART initiation in the first year of life had higher vitamin D levels compared to those on late-ART (18 months to 12 years), independent of age. These results align with our hypothesis that initiating early empiric ART may result in better vitamin D levels, compared to children who were started on ART later after meeting CD4 and clinical criteria, and that these benefits may persist into later childhood.

In this cohort of CALHIV who had received close HIV management and clinical monitoring of health and development for more than a decade, we found that nearly a fifth (18%) had inadequate serum vitamin D. Since impaired vitamin D levels affect skeletal growth [37], our findings suggest clinical providers for CALHIV in the community should consider vitamin D status as part of a comprehensive strategy to optimize child growth and development. Strategies such as advocating for outdoor activities (e.g. sport) to increase sun exposure [38], encouraging consumption of vitamin-D fortified milk, and considering use of vitamin D supplements, are low cost and effective strategies that could improve vitamin D status and health outcomes in CALHIV [39].

A recent systematic review of vitamin D levels in 576 Kenyan children found a mean of 25(OH)D serum concentration of 28.2 ng/mL [40]; similar to the level found in the CALHIV in our study with early-ART initiation (29.5 ng/mL) and higher than in the children who initiated late-ART (22.4 ng/mL). Malnourishment, having dark skin, and having HIV infection are established risk factors for inadequate serum vitamin D levels [15, 16, 41, 42]. We also found that children ≤15 years old were less likely to have inadequate vitamin D levels, consistent with evidence that younger children receive more vitamin D through sun exposure/outdoor activities compared to adolescents [43] and that there is a significant trend in the increase of screen time (either TV, computer or smartphones) among Kenyan adolescents and young adults [44]. Given the age effects on vitamin D level, we adjusted for age and found that the association with early-ART was retained, suggesting that younger age and early-ART are independent factors protective of vitamin D levels.

Oral diseases are highly prevalent in Kenya, and most children do not receive routine primary dental care. Data from the Kenyan Ministry of Health in 2015 found that the overall prevalence of dental caries among children and adolescents (aged 5, 12 and 15 years of age) was 23.9% [45]. We previously reported that caregivers of CALHIV from this cohort reported significantly more oral diseases (42%) compared to HIV-exposed uninfected (27%) and HIV-unexposed children (17%) [46]. Upon examination, the four oral diseases were found to affect 83.3% of children, with dental caries being the most common diagnosis (68.0%). The prevalence of dental caries we found in the early-ART cohort was significantly higher than recently reported in this age group in the general Kenyan population (73% vs. 18%) [45]. Similarly, late-ART cohort had higher prevalence of dental caries compared to 15 year old adolescents from the general population (59% vs. 9%) [45]. These findings demonstrate the high burden of oral diseases among Kenyan CALHIV. Because Kenya has universal health care coverage for children 5 years old and younger (with oral healthcare included), integrating oral health within the regular pediatric medical care could improve the quality of life for this vulnerable population.

While earlier ART initiation has unequivocal benefits for reducing child morbidity and mortality, earlier initiation of ART could have varied effects for different aspects for oral health. Earlier immune restoration in the oral mucosa could normalize salivary IgA, AMPs, and other antimicrobial defenses and *reduce* the risk of dental caries and soft tissue diseases. However, drug exposure during early tooth development can compromise enamel, leading to a *higher* risk of caries. Although this cohort is small, we did find some evidence of tooth compromise associated with earlier ART. We found a trend for higher prevalence of dental caries, dry mouth, enamel hypoplasia and other ulcers (not HSV/aphthous) in the early-ART compared to the late-ART groups. Oral ulcers and dry mouth are often associated with reverse transcriptase inhibitors, and dry mouth can also be present among individuals receiving protease inhibitor therapy [47, 48]. While generally benign, oral ulcers and dry mouth are treatable conditions, and addressing them is another way in which providers could improve quality of life and improve growth and development in CALHIV.

Evidence of the role of vitamin D in the development of oral diseases is unclear with inconclusive reports [49]. In an umbrella of systematic reviews and meta-analysis, vitamin D was identified as a possible preventative agent in the incidence of dental caries (pooled RR = 0.53; 95% CI = 0.43–0.65) [50, 51]. Additionally, a cross-sectional study (2018) found that among the 276 special care needs children, those with suboptimal 25-hydroxyvitamin D were twice as likely to have dental caries compared to children with optimal levels (RR = 2.14; 95% CI = 1.45–3.16) [26]. Vitamin D has been associated to increase production of saliva and alter saliva composition by increasing the amount of calcium ions and pH [7–9]. Furthermore, vitamin D deficiency may decrease the activation of cathelicidins, human defensins, and histatins, salivary AMPs that disrupt the membrane integrity of oral bacteria [52–54] associated with oral diseases [55, 56]. In our study, with and without adjustment of age at the time of oral exam, the risk of dry mouth among children with an inadequate vitamin D level was fairly similar to those who had an adequate vitamin D level. While dental caries is a multifactorial disease, our small sample size may have limited statistical power to find an association. Longitudinal studies will be important provide stronger evidence on the role serum vitamin D in influencing oral diseases.

Our study has many strengths and several limitations to note. Firstly, the inclusion of children with extremely long follow-up (>10 years) from ART initiation allowed us to examine the potential relationship between timing of ART initiation and vitamin D levels much later in childhood. Systematic data collection, a detailed oral examination using WHO-standardized tools, and vitamin D assessments blinded to child ART exposure provide rigor to our approach. The small sample size limited our availability to conduct robust regression analyses. Secondly, the high level of the four oral diseases throughout the whole study population might have precluded us from identifying significant associations regarding vitamin D and oral diseases. Oral examinations were not conducted in a dental unit with ideal illumination and dental isolation (air pressured syringe) limiting opportunities to fully diagnose the four oral diseases. We did not collect data on behavior (sun exposure, diet, and supplements) or consumption of vitamin-D fortified milk, so we cannot adjust for this as a confounder. Because this cohort was accrued from two randomized control trials with different eligibility criteria over different periods of the HIV epidemic; we cannot completely account for confounding by age, immunosuppression, and exposure to maternal ART at enrollment. We also assume that survival bias from the late-ART cohort could affect our estimates of association and limit generalizability of our findings. We do not anticipate that the short, planned treatment interruption at 2 years post-ART would have a significant effect on vitamin D assessment at 10 years post-ART in the randomized children; however, CALHIV frequently have unplanned treatment interruptions and it is possible that longer or more frequent ART interruptions could disrupt vitamin D status. Lastly, since we conducted a cross-sectional study, we were unable to make causal inferences between the timing of ART initiation and occurrence of the four oral diseases among children and adolescents.

In conclusion, this study expands our knowledge on how ART exposure may impact vitamin D levels and the prevalence of the four common oral diseases among children and adolescents in Kenya and suggests providers for CALHIV in Kenya may consider vitamin D status as part of a comprehensive approach to HIV care. Longitudinal studies of large cohorts should be used to assess causal relationships of vitamin D and the prevalence of oral diseases in children and adolescents infected with HIV. Integrating oral health within the continuum care of CALHIV will reduce the burden of oral diseases and increase the quality of life of these children and their families.

## Supporting information

S1 File Inclusivity in global research. (DOCX)
